# Clinical Application of Electrical Impedance Tomography in Emergency and Critical Care Medicine

**DOI:** 10.3390/jcm15103779

**Published:** 2026-05-14

**Authors:** Yoshiaki Iwashita, Satoru Nebuya

**Affiliations:** 1 Head Office for Regional Collaboration and Innovation, Shimane University, Izumo 693-8501, Japan; 2 Department of Emergency and Critical Care Medicine, Shimane University, Izumo 693-8501, Japan; 3 Joint Research in Advanced Medicine for Electromagnetic Engineering, Faculty of Medicine, Shimane University, Izumo 693-8501, Japan; nebuya@posh-wl.co.jp; 4 POSH WELLNESS LABORATORY Inc., Minato 107-0051, Japan

**Keywords:** electrical impedance tomography, mechanical ventilation, functional imaging, bedside monitoring

## Abstract

Electrical impedance tomography (EIT) is a promising imaging tool in critical care. Its capacity to provide noninvasive bedside visualization of regional ventilation and perfusion with high temporal resolution makes it an ideal monitoring modality for patients on ventilation. However, its widespread implementation has been hindered by physical limitations in spatial resolution and a lack of robust evidence linking its use to improved clinical outcomes. In recent years, the commercialization of several bedside devices has led to growing clinical experience, gradually yielding concrete evidence regarding its clinical utility. Furthermore, beyond respiratory monitoring, data are increasingly accumulating in non-pulmonary fields, including perfusion, neuro-critical care and gastroenterology. Therefore, the objective of this review is to synthesize emerging evidence regarding the recent clinical applications of electrical impedance tomography and discuss future perspectives.

## 1. Introduction

**Figure 1:**
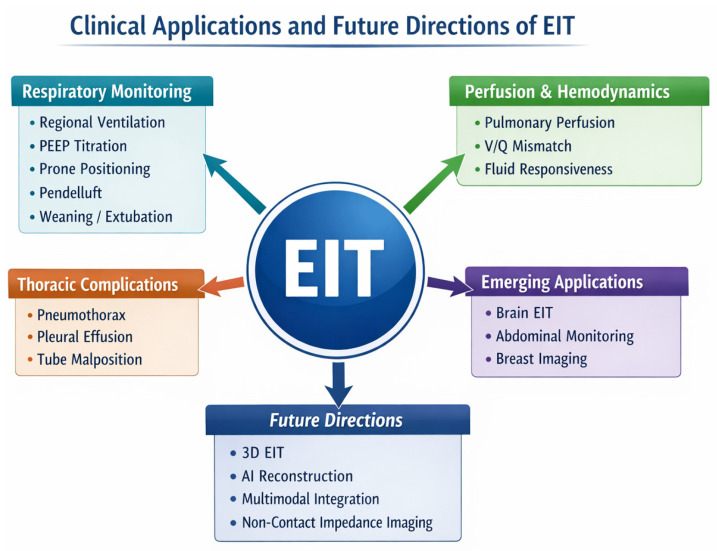
Overview of current and emerging clinical applications of EIT, electrical impedance tomography; PEEP, positive end-expiratory pressure; V, ventilation; and Q, perfusion.

The principles of electrical impedance tomography (EIT) originated in the field of geophysics for subterranean exploration. Its medical application was pioneered in the 1980s at the University of Sheffield by Barber and Brown, who developed the first system capable of generating cross-sectional images of the body using surface electrodes [1]. The first promising clinical application was in the respiratory system because the thorax exhibits the highest natural contrast in the human body. Air is electrically insulating, whereas blood and tissue are conductive. The massive cyclic impedance changes during ventilation allow for robust image reconstruction using time-difference algorithms, making EIT an ideal tool for visualizing regional ventilation distributions without radiation exposure. However, the clinical utility of EIT is not limited to the lungs. Biological tissues exhibit distinct bioimpedance properties based on their physiological state such as fluid content, blood volume, and cellular integrity. Therefore, the objective of this review was to provide a comprehensive overview of EIT as a versatile bedside imaging modality. We explore its established role in respiratory failure, its emerging applications in hemodynamics, neuro-critical care, and abdominal imaging, and critically discuss its strategic positioning relative to the “gold standard” computed tomography (CT) scan (Figure 1).

## 2. Respiratory Monitoring

### 2.1. Why EIT Is Used for Respiratory Monitoring

Clinical application of EIT began with respiratory monitoring, not only because of the physiological nature of EIT but also because of clinical demand. Acute respiratory distress syndrome (ARDS) is characterized by heterogeneous lung injury especially for the tandem axis. A cross-sectional image is required for understanding the pathophysiology of ARDS; however, it is difficult to move the patient to the CT room because of several attached monitoring devices, ventilators, and other invasive devices such as extracorporeal membrane oxygenation (ECMO) and Impella. It is generally believed that positive end-expiratory pressure (PEEP) promotes alveolar recruitment in ARDS; however, determining the imaging-based “ideal” PEEP—i.e., the level that best balances recruitment and overdistension across lung regions—is challenging because real-time cross-sectional imaging is rarely available at the bedside. Consequently, in routine practice, PEEP is often titrated using global physiological surrogates such as respiratory system compliance rather than direct visualization of regional lung mechanics. Portable chest radiography is the most common imaging device used in the intensive care unit (ICU), but it is difficult to assess the tandem axis heterogeneity of the patient’s lung. However, EIT has the potential to visualize cross-sectional lung images at the bedside in a real-time, radiation-free manner. In the 2010s, three companies succeeded in commercializing EIT machines: Dräger, Swisstom (Currently SenTec), and Timpel. Clinical data has been accumulated after that.

### 2.2. Positive End-Expiratory Pressure Titration

PEEP titration is one of the most promising applications for respiratory EIT. Costa et al. proposed an EIT-based method to determine an appropriate PEEP by quantifying regional collapse and overdistension during a decremental PEEP trial [2]. Building upon this physiological framework, recent clinical trials have evaluated whether EIT-guided PEEP titration improves physiological variables and patient-centered outcomes compared with conventional strategies. For instance, a randomized controlled trial (RCT) by Hsu et al. compared an EIT-guided approach balancing collapse and overdistension with the conventional pressure–volume (P–V) curve method in patients with moderate-to-severe ARDS and reported lower selected PEEP and driving pressure at 48 h, along with improved survival in the EIT group [3].

EIT has also highlighted limitations of standardized, “one-size-fits-all” approaches such as the ARDSNet PEEP/FiO_2_ tables [4]. In mechanically ventilated patients with COVID-19-related ARDS, Somhorst et al. reported that EIT assessment suggested clinically meaningful deviations (≥2 cmH_2_O) from table-derived PEEP settings in over 60% of patients, supporting the concept that optimal PEEP may vary across individuals [5]. Beyond observational comparisons, randomized evidence has continued to accumulate. He et al. conducted a randomized controlled clinical trial of early individualized PEEP guided by EIT in ARDS [6]. Jimenez et al. reported in a randomized crossover pilot trial that EIT-guided PEEP titration reduced mechanical power in ARDS [7]. More recently, Mauri et al. evaluated personalized PEEP in spontaneously breathing ARDS patients using simultaneous EIT and transpulmonary pressure monitoring in a randomized crossover trial [8].

A recent systematic review and meta-analysis by Songsangvorn et al. reported that EIT-guided PEEP titration improved lung compliance and reduced driving pressure and mechanical power compared with conventional empirical methods, while interpretation of patient-centered outcomes was limited by heterogeneity in protocols and study designs [9]. Importantly, evidence regarding prognosis remains mixed. Van Trung et al. reported an RCT comparing EIT-guided PEEP with a low PEEP/FiO_2_ strategy in moderate-to-severe ARDS, showing improvements in oxygenation and respiratory mechanics but without definitive mortality benefit [10]. In addition, Maia et al. compared four PEEP titration strategies (EIT, transpulmonary pressure via esophageal catheter, best compliance, and low PEEP/FiO_2_ table) and found no significant difference in mean compliance over the first three days and poor agreement between methods [11]. The EITVent randomized clinical trial demonstrated a non-significant reduction in 28-day mortality compared with a lower PEEP/FiO_2_ table strategy and was terminated early for futility at a preplanned interim analysis [12]. Collectively, these data support EIT as a physiologically informative tool for individualized PEEP selection and for visualizing regional trade-offs not captured by global surrogates; however, whether EIT-guided PEEP improves prognosis likely depends on patient phenotype (e.g., recruitability), protocol standardization, and implementation context, and remains to be clarified.

### 2.3. Visualization and the Treatment of Pendelluft

In spontaneously breathing patients, strong inspiratory efforts can cause rapid redistribution of gas from non-dependent to dependent lung regions before the ventilator-delivered tidal volume is fully distributed—a phenomenon known as “pendelluft.” Yoshida et al. demonstrated that EIT can detect this transient regional gas redistribution in real time, which is typically not apparent from global ventilator signals alone [13]. Subsequent work has advanced from qualitative detection to quantitative assessment, showing that pendelluft can generate substantial regional dynamic stress in dependent lung regions, with local driving pressures markedly exceeding the global driving pressure displayed on the ventilator [14].

From a therapeutic perspective, EIT-based monitoring provides a physiological rationale for interventions aimed at mitigating pendelluft and potentially reducing patient-self-inflicted lung injury (P-SILI). For example, increasing PEEP can stabilize dependent lung units and reduce pendelluft amplitude in selected settings [15]. More recent physiological studies also suggest that pendelluft magnitude and associated expiratory muscle activity during the transition to spontaneous breathing are modulated by ventilatory settings (e.g., pressure support level and PEEP), emphasizing that EIT-derived pendelluft should be interpreted in conjunction with respiratory drive and patient–ventilator interaction [16]. Overall, EIT offers a bedside approach to identify occult regional phenomena and to guide individualized adjustments of ventilatory support, while acknowledging that thresholds and protocols require further validation.

### 2.4. Assessment of Recruitability

A fundamental challenge in ARDS management is that recruitability varies substantially across patients. Applying generic high-pressure recruitment maneuvers (RMs) to a lung with low recruitability risks overdistension of already aerated units and may exacerbate ventilator-induced lung injury (VILI). EIT enables dynamic bedside assessment of regional impedance changes during RMs (e.g., stepwise PEEP elevation), allowing estimation of recruited volume versus overdistension in real time and supporting individualized decisions on whether to proceed with or terminate an RM [17].

Prone positioning is another cornerstone intervention in moderate-to-severe ARDS, and EIT can visualize the redistribution of ventilation toward dorsal (dependent) regions after turning. Prior studies have reported that EIT-derived changes in regional ventilation and/or V/Q-related indices during early prone positioning are associated with oxygenation response and clinical trajectory [18,19]. Importantly, posture can substantially alter chest wall mechanics and regional lung stress, meaning that PEEP titrated in the supine position may no longer be appropriate once prone; continuous EIT monitoring can therefore support reassessment and re-titration of PEEP during prone positioning to maintain dorsal recruitment while avoiding ventral overdistension [20,21].

More recently, a prospective study suggested that early EIT-derived indices may help predict prone responsiveness and identify non-responders who may require earlier reassessment of ventilator settings or alternative strategies [22]. These findings position EIT not only as a monitoring tool but also as a candidate bedside instrument for physiological stratification, while external validation and protocol standardization remain necessary.

### 2.5. Weaning and Extubation Readiness

Beyond PEEP titration and recruitment assessment, EIT has been increasingly explored during spontaneous breathing trials (SBTs) and extubation. Given that it provides continuous visualization of the regional ventilation distribution and end-expiratory lung impedance at the bedside, EIT can detect physiological deterioration during SBTs before overt clinical failure becomes apparent. A recent observational study showed that patients with SBT failure developed greater ventilation inhomogeneity during the trial [23], whereas the VISION study suggested that an increased ventral-to-dorsal ventilation difference early during SBT was associated with liberation failure [24]. In difficult-to-wean patients, pendelluft detected using EIT during T-piece trials was associated with prolonged ventilation and higher 28-day mortality [25]. These findings suggest that EIT may complement conventional weaning indices by adding real-time regional information on respiratory mechanics and patient self-inflicted lung stress, although larger validation studies are needed.

Recent physiology-oriented work has also examined stepwise reductions in pressure support and the balance between over-assistance and under-assistance, using EIT-derived regional ventilation distribution together with indices of respiratory drive and muscle effort [26]. Collectively, these findings suggest that EIT may complement conventional global weaning indices by adding regional physiological information; however, larger prospective validation studies are required to define clinically actionable thresholds and to confirm whether EIT-guided weaning strategies improve patient-centered outcomes.

## 3. Imaging of Pulmonary Perfusion and Hemodynamics

### 3.1. Pulmonary Perfusion

Historically, EIT has been viewed almost exclusively as a ventilation monitor. However, evaluating true gas exchange requires the mapping of both ventilation (V) and perfusion (Q). The most established EIT perfusion technique relies on the indicator dilution principle of injecting a bolus of hypertonic saline (a highly conductive contrast agent) through a central venous catheter during a brief breath-hold. The transit of this bolus causes a transient drop in impedance, outlining the pulmonary vascular bed [27]. More importantly, this technique is no longer restricted to research settings. Modern commercially available bedside EIT devices (e.g., Timpel) are equipped with dedicated software modules that automatically synchronize impedance changes and instantly generate bedside V/Q mismatch maps.

However, apneic pauses may be poorly tolerated by patients with severe ARDS. Victor et al. recently described a first-pass kinetic model for saline-contrast EIT, which enables pulmonary perfusion estimation during uninterrupted breathing [28]. Their letter showed good agreement between the no-apnea approach and the conventional apnea-based method, suggesting a feasible alternative for bedside perfusion mapping.

The clinical application of perfusion EIT may facilitate rapid bedside recognition of pulmonary perfusion defects including pulmonary embolism (PE). In such cases, EIT can reveal the characteristic patterns of ventilated but non-perfused lung regions, consistent with increased dead space [29,30,31]. Beyond diagnosis, EIT-derived V/Q mismatch appears to have prognostic significance. Spinelli et al. reported that unmatched ventilation and perfusion, measured using EIT, were independently associated with mortality in patients with ARDS [32]. During the COVID-19 pandemic, Mauri et al. showed that patients with COVID-19 ARDS often exhibited substantial V/Q mismatch, with ventilated non-perfused regions predominating over perfused non-ventilated regions in most evaluated cases [33]. These findings support the hypothesis that pulmonary vascular dysfunction contributes to hypoxemia in a subset of patients.

### 3.2. Fluid Responsiveness and Non-Contrast Heart–Lung Interactions

In addition to contrast-based pulmonary perfusion imaging, EIT has been explored for noninvasive hemodynamic monitoring. By separating cardiac-related impedance oscillations from the dominant respiratory signal, thoracic EIT may provide continuous information on cardiac activity and, possibly, stroke volume [34]. Experimental work has further shown that the EIT-derived stroke volume variation (SVV) can be calculated automatically from cardiac-related impedance changes and may reflect the dynamic indices of fluid responsiveness, although its accuracy is influenced by the type of lung injury [35]. Thus, by simultaneously tracking ventilation-related lung distension and cardiac-related impedance signals during positive-pressure ventilation, EIT has the potential to evolve into a more integrated cardiopulmonary monitoring tool; however, clinical validation for the routine assessment of fluid responsiveness remains limited [34,35].

## 4. Detection of Thoracic Complications

### 4.1. Pneumothorax

Air in the pleural space produces a characteristic high-impedance region on thoracic EIT images. Continuous cross-sectional monitoring may facilitate the early detection of pneumothorax, including during mechanical ventilation, before overt hemodynamic deterioration occurs [36].

### 4.2. Pleural Effusion

Pleural effusion is associated with characteristic out-of-phase impedance changes on EIT, particularly in dependent lung regions. Becher et al. showed that these changes were markedly greater in patients with pleural effusion and decreased significantly after drainage, suggesting that EIT may be useful for bedside monitoring of pleural fluid accumulation and response to thoracentesis [37].

### 4.3. Endotracheal Tube Placement

EIT can provide rapid bedside information on right-left ventilation distribution and may help identify endobronchial intubation or malposition of double-lumen tubes by demonstrating marked asymmetry or absence of ventilation in one lung. However, it should be regarded as an adjunct rather than a replacement for fiberoptic bronchoscopy when precise airway confirmation is required [38,39].

## 5. Neuro-Monitoring: Brain EIT (Current Challenges and Future Frontiers)

### 5.1. The “Time Is Brain” Paradigm and EIT’s Potential

In acute stroke care, rapid differentiation between ischemic and hemorrhagic stroke is essential, particularly before intravenous thrombolysis. Mobile stroke units (MSUs) equipped with onboard imaging can shorten treatment delays and improve prehospital triage; however, broader implementation remains constrained by cost and logistical complexities [40]. In principle, brain EIT could provide a portable adjunctive approach for prehospital stroke assessment because ischemic and hemorrhagic lesions are expected to differ in their electrical properties [41]. Furthermore, recent preclinical studies have introduced contrast-enhanced electrical impedance tomography perfusion (EITP) to directly visualize cerebral hemodynamics. For instance, in an experimental rabbit model, EITP successfully mapped regional cerebral perfusion deficits and detected acute ischemic stroke within 5 min of onset [42]. A preceding pilot study using a rabbit internal carotid artery occlusion model demonstrated that contrast-enhanced EIT could visualize cerebral perfusion patterns after intra-arterial glucose injection, supporting the feasibility of impedance-based cerebral perfusion imaging before its application to ultra-early stroke detection [43]. These ultra-early detection capabilities highlight the potential of contrast-enhanced approaches for rapid triage in the hyperacute phase of stroke.

### 5.2. The Anatomical Barrier and the Multi-Frequency Frontier

Despite its promise, brain EIT has not yet entered routine clinical practice. A major challenge is the high resistivity of the skull that reduces current penetration into the brain and promotes current shunting through superficial tissues [44]. Therefore, multifrequency EIT (MFEIT) has been investigated as a possible strategy for stroke assessment because frequency-dependent tissue properties may help distinguish lesion types without relying entirely on a prior baseline image. However, robust human imaging and classification methods are still under development [45].

### 5.3. Investigational Uses in the ICU

In the ICU, brain EIT has also been explored as a noninvasive monitoring tool for cerebral edema. In patients with cerebral hemorrhage undergoing dehydration therapy, EIT-derived impedance changes correlate with simultaneously measured intracranial pressure trends, suggesting a potential value for bedside trend monitoring rather than definitive ICP replacement [46].

## 6. Gastrointestinal Imaging

### 6.1. The Clinical Dilemma of Gastrointestinal Monitoring

Gastrointestinal dysfunction, ranging from feeding intolerance and ileus to acute mesenteric ischemia, is common in critically ill patients and associated with worse outcomes and higher mortality. Despite its clinical importance, continuous bedside monitoring of the abdominal function remains limited [47]. Current diagnostic approaches are often imperfect in unstable ICU patients. Endoscopy is invasive, whereas abdominal CT may be constrained by the need for transport, contrast administration, and the patient’s hemodynamic condition.

### 6.2. Monitoring Gastric Emptying and Enteral Feeding

In this context, abdominal electrical impedance-based monitoring has been explored as a noninvasive approach for functional gastrointestinal assessment. One of the earliest proposed applications was the evaluation of gastric emptying [48]. Changes in abdominal impedance have been used to track the distribution and emptying of gastric contents during enteral feeding, suggesting a potential value for bedside assessment of feeding tolerance and gastric residual dynamics.

### 6.3. Technical Challenges and the Path Forward

Despite this potential, abdominal applications remain far less developed than thoracic EIT. In contrast to the thorax, abdominal signals are weaker and less regular because gastrointestinal motility is slow, intermittent, and spatially heterogeneous. In addition, bowel gas, respiratory motion, and changing abdominal geometry can substantially distort impedance measurements. Future progress will depend on improved signal-processing methods and validation studies that can better isolate gastrointestinal activity from respiratory- and motion-related artifacts.

## 7. Technical Challenge and Future Direction

Despite substantial technical progress, the wider implementation of EIT still depends on standardization, automation, and integration into the clinical workflow. Recent narratives and expert reviews have highlighted the persistent challenges related to electrode positioning, reconstruction algorithms, interpretation of derived indices, and interdevice variability. Moreover, whereas CT utilizes X-rays that travel in straight lines as a “hard-field” modality and therefore provides high spatial resolution, EIT is a “soft-field” technique in which injected current propagates diffusely, inherently limiting spatial resolution and anatomical precision [49]. Therefore, EIT should not be regarded as a direct substitute for X-ray-based CT or other high-resolution anatomical imaging modalities. Rather, its principal clinical value lies in situations that require continuous, radiation-free, real-time functional monitoring such as tracking regional ventilation, perfusion, and physiological responses to therapeutic interventions at the bedside. In this sense, CT and EIT should be viewed as strategically complementary rather than competing technologies: CT provides definitive anatomical “snapshot” information, whereas EIT offers dynamic trend monitoring and repeated functional assessment over time.

The absence of ionizing radiation broadens the potential deployment of impedance-based monitoring beyond conventional radiology environments. In the future, EIT may become increasingly relevant not only in hospitals but also in ambulances, emergency transport systems, and other point-of-care or non-radiology settings where radiation shielding is impractical. This portability may be particularly valuable for screening, triage, serial reassessment, and treatment monitoring when immediate access to CT is limited. This concept is particularly attractive in prehospital and resource-constrained environments, although further validation is required for each specific indication.

### 7.1. Three-Dimensional EIT

A major future research direction is the transition from conventional two-dimensional belt-based monitoring toward three-dimensional EIT. Most currently available systems acquire a single transverse slice, which does not fully capture the craniocaudal heterogeneity. Recent work, including an initial clinical report on multi-slice 3D EIT [50], suggests that volumetric impedance imaging is beginning to move from technical development toward early clinical application.

### 7.2. AI and Machine Learning

Another important frontier is AI-assisted reconstruction. Deep learning approaches, including D-bar-based neural network methods, are being developed to mitigate the classical inverse problem, improve robustness to noise, and enhance structural image quality beyond that achievable with traditional reconstruction alone [51]. These methods remain under active development, and they represent a plausible route toward more automated and clinically interpretable EIT systems.

### 7.3. Multimodal Integration

Future research should focus on multimodal integration. Rather than functioning in isolation, EIT may become more powerful when combined with complementary modalities and clinical data streams such as CT, ultrasound, arterial blood gas analysis, ventilator waveforms, and hemodynamic monitoring. In this integrated framework, EIT can add continuous regional functional information that static anatomical imaging cannot provide, thereby supporting individual decision making.

### 7.4. Non-Attached EIT

A future direction may be the development of non-contact impedance-based tomographic imaging. Conventional EIT requires surface electrodes to be attached directly to the body, and stable measurement depends on minimizing the influence of skin–electrode impedance. By contrast, the newly proposed approach applies a weak alternating current in space and detects the resulting subtle magnetic field changes to estimate the conductivity and generate tomographic images [52]. Relatedly, capacitively coupled EIT has been investigated for brain imaging as an approach that reduces the dependence on direct electrode–skin contact, suggesting a broader technical trend toward less contact-dependent impedance imaging [53]. If such technologies become clinically practical, it may enable complete noncontact tomographic imaging, potentially even through electrically insulating barriers. This concept can substantially broaden the applicability of impedance-based imaging beyond conventional electrode-dependent monitoring.

## 8. Conclusions

Electrical impedance tomography is a promising bedside imaging modality that provides a continuous, radiation-free assessment of regional physiological functions. Among the current applications, respiratory monitoring is the most widely used, with established usefulness in the evaluation of regional ventilation distribution and growing roles in PEEP titration, prone-position management, recruitability assessment, and weaning support. Other applications, including perfusion imaging, hemodynamic monitoring, neuromonitoring and abdominal imaging, remain promising but are generally less validated and should currently be regarded as adjunctive or investigational. Importantly, EIT is not intended to replace conventional high-resolution imaging modalities, such as CT. Instead, its clinical value lies in repeated functional monitoring at the bedside, particularly in situations in which dynamic physiological changes and treatment responses must be monitored over time without radiation exposure. Further progress will require standardization of the methodology, improved reproducibility across devices and centers, automated and interpretable reconstruction strategies, and stronger outcome-based clinical evidence. With ongoing advances in three-dimensional imaging, artificial intelligence, multimodal integration, and emerging non-contact impedance-based technologies, the role of impedance-based imaging may continue to expand in the point-of-care era.

## Data Availability

No new data were created or analyzed in this study.

## Abbreviations

The following abbreviations are used in this manuscript:

EIT	electrical impedance tomography
ARDS	acute respiratory distress syndrome
PEEP	positive end-expiratory pressure
ECMO	extracorporeal membranous oxygenator
MFEIT	multi-frequency electrical impedance tomography
MSU	mobile stroke unit
SVV	stroke volume variation
